# Risodontrypy; or Treatment of Exposed Nerves

**Published:** 1853-04

**Authors:** C. O. Cone

**Affiliations:** Baltimore


### Selected Articles.

*Risodontrypy; Or, Treatment of Exposed Nerves.*—BY C. O. CONE, M. D., of Baltimore.

[The following cases of exposed Dental Nerves, were treated by *Risodontrypy*, or Hullihen's Operation, and are copied from notes taken at the time of the operation, by Dr. Cone.]

CASE 1st. *Sept.* 1*st*, 1852.—While excavating a carious cavity on the posterior aproximal surface of the left inferior lateral incisor for Mrs. R., aged about 22 years, of a nervous sanguine temperament, and general good health, I exposed the nerve-pulp at a small fissure of the superior portion of the nerve chamber. The touch of the nerve was attended with acute pain, and the exhaustion of the atmosphere from the cavity by the tongue and lips of the patient was attended with a sharp pain.

I endeavored to penetrate the fang of the tooth just below the terminal margin of alveolus with a bow-drill, but the refractory character of my patient demanding my left arm and hand to control the head, together with a want of dexterity in the use of this instrument in the mouth, compelled me to complete the operation with a drill made of an ivory handled excavator. The drill was conveyed through the fang of the tooth until its entrance into the nerve chamber, and which was very distinctly felt. The operation was attended with but little pain after the gum was puctured, until the drill entered the nerve-cavity, when the patient uttered an exclamation of pain. Immediately after the operation no pain was felt in the tooth, and no sensation of pain could be produced by exhausting the air from the cavity in the crown of the tooth. I proceeded to fill the excavated cavity at once.

*Sept.* 3*d*.—Saw Mrs. R. She has not experienced any pain in the tooth treated by Hullihen's operation. She says it felt a little sore, but not elongated. I could detect no change of complexion of the crown of the tooth. Some inflammation existed in the gum, most of it dependent on the presence of drill cuttings. Removed such as I could.

*Sept.* 8*th*.—I examined the tooth in Mrs. R's. mouth, operated on by Hullihen's method. I could detect no change of complexion of the crown of the tooth indicating the loss of its vitality. No inflammation in the gum. The opening made by the puncture of the drill nearly closed. Patient has felt no pain in the tooth since the operation. This excited my suspicion whether the drill had not amputated the nerve. Endeavored to test the vitality of the tooth by galvanism. Took a small strip of commerce zinc, and brought it in contact with the tongue and the gold plug of the tooth operated on. No sensation indicating vitality was felt. Touched other gold plugs in the mouth plugged in cavities, which were sensitive at the time the cavities were filled. In only one of these teeth was any pain experienced when the plug was touched with the zinc.

This time the setting of the patient was employed in polishing the filed surface and the plug introduced in the tooth having suffered the operation of Risodontrypy. The polishing material was conveyed on a piece of wood, which passed readily between the tooth operated on and its neighbor. The heat produced by the friction of the wood and polishing material on the above named tooth was so marked as to leave no doubt of the vatality of the crown of the tooth. Care was exercised to avoid deception, and secure the neighboring tooth from contact with the stick and polishing material.

*Oct.* 15*th.*—I examined the tooth treated by Hullihen's operation, prior to Mrs. R. leaving the city for the Pacific coast. The puncture through the gum was entirely closed. She has felt no uneasiness except occasionally on taking very cold water a momentary pain was felt in the tooth, but nothing that could be called discomfort.

Case 2d. *Sept.* 3*d*, 1851.—To-day I exposed the nerve of the first right inferior molar on the posterior aproximal surface, for Miss W., aged about 22, of a nervous sanguine temperament, and of a scrofulous diathesis.

The superior and posterior portion of the crown was so removed by the file as to afford free vision of the cavity. The nerve was exposed at two distinct points corresponding to the tubercles of the posterior grinding surface of the tooth. After the cavity was prepared, the exhaustion of the air from the cavity gave great pain. The introduction of the end of the tongue into the cavity produced the same effect. In excavating the diseased bone from the cavity the nerve and its vessels had been wounded, and the blood flowed freely into the cavity. The patient was now dismissed for one hour, with the nerve protected by the cavity being filled with beeswax. When she returned, she complained of a violent tooth-ache,—

I punctured the tooth with a "fixed-drill." The drill was placed just at the margin, but above the alveolus, and entered the nerve chamber at the neck of the tooth, just above the division of the fangs. The entrance of the drill into the nerve cavity was distinctly felt by its plunge. The operation was attended by a good deal of pain;—the patient exclaiming "that's worse than having a tooth extracted." I could produce pain on applying pressure to the nerve after the operation was performed, and by the same means increase the flow of blood from the cavity formed at the neck of the tooth. The blood that flowed from this cavity was arterial. The pain that was before felt on exhaustion of the air from the cavity, was no longer felt when the same test was applied. The pain felt by the patient before the operation gradually subsided, and in fifteen minutes no pain was felt in the tooth. The only discomfort experienced was the "smarting of the gum."

The want of confidence growing out of experience in this class of practice, together with the violent symptoms which preceded the operation, caused me to hesitate in relation to the treatment of the tooth. I finally determined to introduce into the cavity a temporary filling, and if the operation promised success, replace the tin with gold. I accordingly filled the tooth with block-tin foil.

*Sept.* 4.—Saw Miss W. again. No pain has been felt in the tooth, that could be called tooth-ache. The tooth did not feel elongated, but she complained of soreness and pressure on the gum. Examined the tooth, no uneasiness could be produced by pressure applied to the crown of the tooth in any direction. Above the puncture, or between the puncture and the neck of the tooth, the circulation was stopped in the gum by the accumulation of the drill cuttings; removed them, which offered relief to the pressure of the gum.

*Sept.* 13.—Miss W. called to inform me that the gum and parts about the tooth had been quite painful at night for the last week. The pain not of a throbbing or lancinating character but dull and heavy. Pain felt more through the night than during the day. Not sufficiently severe to disturb sleep. On examining the tooth found the gum a good deal inflamed on the labial portion of the tooth where the drill punctured it, soreness was felt on this side of the tooth when pressure was applied with the finger down the alveolus to near the termination of the fang of the tooth. Could apply pressure on the lingual portion of the gum without any pain. Violence applied in all directions to the crown of the tooth produced no pain, except when an instrument was forced between the tooth operated on and its posterior neighbor. The file had been used boldly between these two teeth and had lacerated the periosteum where it unites with the free edge of the gum. Patient avoids mastication on this side of the mouth. No elongation of the tooth.

*May* 17, 1852.—I had an opportunity of removing the tin plug. On examination of the tooth no soreness was felt by pressure on either the gum or the tooth. No marks of the puncture through the gum, which is now healthy about the neck of the tooth. Patient says the soreness of the gum continued some weeks and then entirely abated.

On removing the temporary plug I could find no cavity leading into the nerve chamber. I could pass a steel excavator over the whole floor of the cavity, it being met in all directions with a bony resistance. To the eye the floor of the cavity appeared to be covered at the points where the nerve had been exposed, with bone of a darker hue, bu twhich presented a firm resistance. The injection of ice water into the cavity gave acute pain which soon subsided. Proceeded to plug the tooth with gold at this sitting.

*June* 30.—While operating on some other teeth for Miss W., examined the tooth treated by Hullihen's operation and can see no diseased action, and the patient experiences no change marking it as being different from teeth not having suffered the operation.

Case 3d. *Sept.* 4. 1851.—Mr. W., aged about 19, of a nervous bilious temperament, consulted me in relation to a cavity on the bucal and masticating surface of the inferior, right, first molar. The tooth had been filled when the cavity was confined to the bucal surface of the tooth, but failed. The operator in endeavoring to shape the cavity to retain the filling on the second trial removed so much dentine as to leave insufficient support on the masticating surface of the tooth, and this portion of the wall of the cavity was broken down. In endeavoring to obtain sufficient depth &c. to retain a plug in this position, with sufficient firmness to resist mastication, I exposed the nerve at a point corresponding to the anterior labial tubercle of the masticating surface of the tooth. The exposure was slight and could be detected by the excavator dipping into a fissure and producing a nervous shock. The vessels of the nerve were not wounded so as to bleed, neither could the eye detect the actual exposure of the nerve.

The exhaustion of the atmosphere from the cavity was not attended with much or any pain. The dentine immediately about the fissure which my instrument dipped into, had translucent lines, giving it a coronated-like appearance. Not being fully satisfied that the nerve was fully exposed and caring to drive my excavator unnecessarily into the nerve to test this point, I plugged the tooth temporarily with block-tin foil.

The patient returned after about two hours, complaining that the tooth was very painful and that the pain commenced soon after he was dismissed and had gradually increased. Considering this sufficient proof of the exposure of the nerve, I proceeded to perform Hullihen's operation, with the instrument, and in the manner described in Miss W.'s case. The instrument was distinctly felt to plunge into the nerve cavity. The pain attending the operation was not great. The pain experienced on his return and before the operation, entirely subsided in ten minutes after the operation. It was impossible for me to fill the tooth permanently at this time in consequence of other professional engagements.

*Sept.* 8.—I removed the temporary tin plug from Mr. W.'s tooth. The point at which I supposed the nerve to be exposed was sensitive on the touch of a fine excavator. The exhaustion of the atmosphere from the cavity produced no pain. The tooth had given no pain after the operation, and in answer to my inquiries relative to its condition he replied "it feels sore." No elongation of the tooth. No uneasiness induced by pressing the crown of the tooth in any direction. The gum, at the point where the drill punctured it, was hard and slightly swollen. I plugged the tooth with gold. No pain was felt in condensing this large plug which required a good deal of violence.

*Sept.* 15.—Again examined the tooth of Mr. W. Complains of some pain about or in the tooth at night and in the morning. Pain not violent—not throbbing—not lacerating, but dull heavy pain. Gum swollen and tender on labial portion of the tooth, pressure applied on the crown of the tooth, in that direction was attended with pain. Mastication is not performed on this side of the mouth.

*Sept.* 25.—Mr. W. informed me that his tooth has been improving, the soreness of the gum has almost or quite subsided. Some days ago he endeavored to eat a cold apple, which was brought in contact with the tooth, which produced pain that continued for some hours. The puncture through the gum can not be detected; pressure in any direction gives the patient no sensation of pain.

*Oct.* 1.—Saw Mr. W.—Ice-water applied to the plug of the tooth treated for exposed nerve, gives acute pain. Gum has a healthy appearance. Can masticate on this tooth without pain.*

CASE 4th. *Sept.* 10, 1851.—To-day I exposed the nerve at two distinct parts in excavating a cavity on the posterior approximal surface of the right superior first bicuspis of Miss F., aged about 24, of a bilious nervous temperament of general good health. The exposure of the nerve was attended with a good deal of hemorrhage from its vessels, but no pain to the patient, and when informed that the nerve was exposed, expressed surprise. I performed Hullihen's operation.

*July* 24*th*, 1852.—To-day I have seen Miss F., who resides some 70 miles from the city. The puncture in the gum has not closed, on introducing a bristle into it could detect puss. The patient gave the following history of the tooth: About a month after the operation, the tooth began to be painful on change of temperature of the mouth—in taking first cold, and afterwards warm drinks—finally slight elongation was first experienced, and still later a "pimple" appeared filled with puss where the drill punctured the gum, which she opened. Since that time she has had no pain in the tooth of any kind, but occasionally the "pimple appears, filled with matter." It should be remarked that the first superior, left bicuspis, first and second superior, left molars, and the first and second right, superior bicuspis, first and second, right molar, had lost their crowns from dental caries, but at no time had their destruction been attended with pain. I extracted the fangs of these teeth during my attendance on the patient last September.

CASE 5th. *Sept.* 27, 1851.—Mr. C. of New Orleans, aged about 30, of nervous bilious temperament, and in the enjoyment of general good health, consulted me in relation to the first, right inferior bicuspis, which had been plugged on its anterior approximal surface. The plug had fallen out. The tooth had given no pain. The excavation of the cavity gave not much pain, but yet it was sensitive to the use of the

---

* *Oct.* 20, 1852.—To-day I had an opportunity of filling a small cavity on the masticating surface of the right inferior first molar, for Mr. W. This tooth was treated by Hullihen's operation, on the 4th of Sept., 1851. In excavating this tooth cavity to-day, for the plug, the dentine of the tooth was very sensitive. No indication of disease beyond the carious cavities which were filled, marked the condition of the organ or surrounding parts.

excavator. I exposed the nerve at a small point, and blood flowed from its vessels. Performed Hullihen's operation on the tooth. This was not attended with much pain. After the operations some sensation could be felt on exhausting the atmosphere from the cavity, but not that pain which attended the same effort before the performance of the operation. The nerve is still sensitive to the touch of an instrument when brought in contact with it. No pain was felt, however, on the introduction of the plug.

*Oct.* 6.—Saw Mr. C. to-day. He had felt no symptom in the tooth operated on, marking it from any other tooth in the mouth. The tooth is not sensitive to pressure. No inflammation in the gum, the puncture through the same by the drill cannot be detected by the eye. Mr. C. leaves for New Orleans to-morrow.

*July* 20, 1852.—Had an opportunity of examining the tooth of Mr. C. It presents every appearance of vitality. Gum is healthy about the tooth. No marks of the puncture except a perceptible thickening of the alveolus at just the point where the puncture was made. The patient says he has experienced no sensation in the tooth which would direct his attention to it, and that "the tooth now feels like a live tooth."

CASE 6th. *Sept.* 30.—To-day I exposed the nerve in excavating a cavity on the anterior approximal surface of the superior left central incisor, for Master H., aged 14, of a bilious nervous temperament. The portion of the nerve exposed was not large. No blood followed. Performed Hullihen's operation. The exhaustion of the atmosphere from the cavity after the operation, gave no pain, which was acute when a vacuum was made in the cavity before the operation.

*Oct.* 1.—Master H. does not complain of the central incisor operated on, nor does he feel any change in the tooth, and only a little soreness of the gum. Tooth presents a healthy appearance,—little or no inflammation about the gum. The tooth is not sensitive to pressure applied in any direction.

*Oct.* 3.—Master H. complains of occasional pain being felt about the tooth. Pressure on the crown gives a slight sensation of pain or rather uneasiness. His gum slightly inflamed about the puncture.

*Oct.* 27.—Master H. informs me to-day that the pain and soreness of the incisor operated on have subsided. No uneasiness or discomfort is experienced. Puncture through the gum has closed. No inflammation in the soft tissues. The crown of the tooth shows the complexion of vitality.

*Feb.* 6, 1852.—Master H. called to-day in relation to other teeth, and I examined the incisor operated on for exposed nerve. It has every indication of health. Has not given the patient any uneasiness since October. No mark of the operation to be seen about the gum.

Case 7th. *Oct.* 7, 1851.—Mr. R., of a sanguinous nervous temperament, and general good health, aged about 19, desired me to fill the anterior approximal surface of the second right, superior bicuspis. In removing the diseased dentine from the cavity, the nerve was exposed, and its vessels bled freely The exposure of the nerve was attended with considerable pain. I performed Hullihen's operation. In doing this, the very point of the cutting edge of the drill broke off. I think this occurred at the time the drill entered the nerve cavity. No pain, after the operation, was felt on exhausting the atmosphere from the cavity. The nerve was, however, sensitive to the touch. Plugged the tooth, with expectation of a failure. No pain was felt in the tooth after it was plugged.

*Oct.* 8.—Mr. R. called to let me examine the bicuspis operated on yesterday. No pain has been experienced. Pressure in any direction on the crown of the tooth gives no pain. There is but little inflammation in the gum. Mr. R. leaves this day for Europe.

[TO BE COTINUED.]

